# A Proteomic View to Characterize the Effect of Chitosan Nanoparticle to Hepatic Cells: Is Chitosan Nanoparticle an Enhancer of PI3K/AKT1/mTOR Pathway?

**DOI:** 10.1155/2014/789591

**Published:** 2014-03-16

**Authors:** Ming-Hui Yang, Shyng-Shiou Yuan, Ying-Fong Huang, Po-Chiao Lin, Chi-Yu Lu, Tze-Wen Chung, Yu-Chang Tyan

**Affiliations:** ^1^Instrument Technology Research Center, National Applied Research Laboratories, Hsinchu 300, Taiwan; ^2^Translational Research Center, Kaohsiung Medical University Chung-Ho Memorial Hospital, Kaohsiung 807, Taiwan; ^3^Department of Medical Research, Kaohsiung Medical University Chung-Ho Memorial Hospital, Kaohsiung 807, Taiwan; ^4^Department of Obstetrics and Gynecology, Kaohsiung Medical University Chung-Ho Memorial Hospital, Kaohsiung 807, Taiwan; ^5^School of Medicine, College of Medicine, Kaohsiung Medical University, Kaohsiung 807, Taiwan; ^6^Department of Medical Imaging and Radiological Sciences, Kaohsiung Medical University, Kaohsiung 807, Taiwan; ^7^Department of Nuclear Medicine, Kaohsiung Medical University Chung-Ho Memorial Hospital, Kaohsiung 807, Taiwan; ^8^Department of Chemistry, National Sun Yat-sen University, Kaohsiung 804, Taiwan; ^9^National Sun Yat-sen University-Kaohsiung Medical University Joint Research Center, Kaohsiung 807, Taiwan; ^10^Department of Biochemistry, College of Medicine, Kaohsiung Medical University, Kaohsiung 807, Taiwan; ^11^Department of Biomedical Engineering, National Yang-Ming University, Taipei 112, Taiwan; ^12^Center of Biomedical Engineering and System Biology, Kaohsiung Medical University, Kaohsiung 807, Taiwan

## Abstract

Chitosan nanoparticle, a biocompatible material, was used as a potential drug delivery system widely. Our current investigation studies were the bioeffects of the chitosan nanoparticle uptake by liver cells. In this experiment, the characterizations of chitosan nanoparticles were measured by transmission electron microscopy and particle size analyzer. The average size of the chitosan nanoparticle was 224.6 ± 11.2 nm, and the average zeta potential was +14.08 ± 0.7 mV. Moreover, using proteomic approaches to analyze the differential protein expression patterns resulted from the chitosan nanoparticle uptaken by HepG2 and CCL-13 cells identified several proteins involved in the PI3K/AKT1/mTOR pathway. Our experimental results have demonstrated that the chitosan nanoparticle may involve in the liver cancer cell metastasis and proliferation.

## 1. Introduction

Biomaterials play important roles in regenerative medicine, tissue engineering, and drug delivery [1]. Nanomedicine is the application of nanotechnology in medicine, which enabled the development of nanoparticle therapeutic carriers. These drug carriers are targeted to tumor cell surfaces through the enhanced permeability and retention effect; thus, they are very suitable for the chemotherapeutics delivery in cancer treatment. Nanomaterials have increased surface to volume ratio compared with their bulk materials, and this may confer interesting properties, such as increased mechanical strength. Their distinct physicochemical characteristics, obtained by changing the size and shape, are very different from their natural materials and thus granting new possibilities [2]. Different nanomaterials have various effects on cells. For example, the uptake of silver nanoparticles caused cell proliferation inhibition in mouse leukaemic monocyte macrophage cells [3] and human keratinocytes [4]. In addition, low concentration of gold nanoparticles resulted reduced cell proliferation in rat pheochromocytoma cells and human umbilical vein endothelial cells [5]. On the other hand, single-wall carbon nanotubes (SWCNTs) were investigated for biomedical applications and showed no negative effect for cell proliferation [6].

Chitosan, a biocompatible and biodegradable polymer, is a modified natural carbohydrate polymer prepared by the partial N-deacetylation of chitin (primary unit: 2-deoxy-2-(acetylamino) glucose). Chitosan and chitin, next to cellulose, are the second most plentiful nature and nontoxic, biodegradable cationic polymers. It is a natural biopolymer derived from crustacean shells such as krill, shrimps, lobsters, and crabs [1]. As such, chitosan is an abundant natural polymer available from a renewable resource. Chitosan, a mucopolysaccharide having structural characteristics similar to glycosamines, is a linear *β*(1 → 4)-D-glucosamine and acetyl-*β*(1 → 4)-D-glucosamine, which can be obtained by alkaline N-deacetylation derivative of chitin [7]. Thus, chitosan is usually not a homopolymer of D-glucosamine but a copolymer containing less than 40% N-acetyl-D-glucosamine residues. Chitosan has both reactive amino and hydroxyl groups, which can be used to chemically alter these properties under mild reaction conditions. Therefore, there are many interesting chitosan derivatives, especially for biomedical applications [1, 8, 9]. Chitosan has been proposed for the development of membranes and fibers of hemodialysis and blood oxygenators, skin substitute, wound dressing and suture materials intended for immobilization of enzymes and cells to bind with bile and fatty acids and as a vehicle for drug and gene delivery [10–14]. It has been widely used in several fields of developing treatments as diverse as lung surfactant additives, wound healing, and tissue engineering. Although chitosan is suitable for medical applications, for those applications that involve blood contact such as hemodialysis membranes, chitosan promotes surface induced thrombosis and embolization [10, 12]. It is indicated in the literature that chitosan has the capacity to activate both complement and blood coagulation system [15, 16]. Chitosan is also a bioactive carbohydrate polymer and potentially useful in tissue engineering and for gene and drug delivery [17, 18]. However, none is without disadvantages. For example, chitosan may prevent the absorption of needed vitamins and minerals. It may also be dangerous to those who are allergic to shellfish.

Chitosan nanoparticle prepared by ionotropic gelation technique was first reported by Calvo et al. [19]. It can be formed with sodium tripolyphosphate. The chitosan nanoparticles have gained more attention as drug delivery carriers because of better stability, low toxicity, simple and mild preparation method, and providing versatile routes of administration [20]. Chitosan nanoparticles accumulation typically occurs around the defect area in cells and tissues by hydrophobic interactions. In addition, several physiochemical characteristics of chitosan nanoparticles, such as abilities to cross biological barriers, to protect macromolecules from degradation and to deliver a compound at a target site, were examined as favorable [21].

The development of nanoparticulate drug carriers has followed several routes depending on the final application. Although a wide variety of systems have been designed with their own advantages and limitations, the common goal is to rationalize drug delivery to enhance the bioavailability of the drugs towards targeted diseased cells, promoting the required response while minimizing side effects. Therefore, to use chitosan derivatives for biomedical applications, a test for evaluating biocompatibility must be performed.

In this study, we investigated various methods to analyze and characterize the parameters that influence the uptake of cells on chitosan nanoparticle. The CCL-13 and HepG2 cells were served as cell models for the uptake of chitosan nanoparticles. For instance, using colorimetric techniques such as LDH or BrdU assay is a convenient method and typically applied to many biomaterial researches. Characterizations of chitosan nanoparticles were observed by TEM, particle sizer, and zeta potential. To evaluate early responses of CCL-13 and HepG2 cells to chitosan nanoparticles, a mass spectrometry-based profiling system was adopted for assessing characteristic proteins that were expressed due to the interactions of CCL-13 and HepG2 cells with chitosan nanoparticles. Through the investigation, various proteins that influence the early responses of CCL-13 and HepG2 cells on chitosan nanoparticle were found, and, as we know, some of them have not been reported in the study of cell-nanoparticle interactions.

## 2. Materials and Methods

### 2.1. Chitosan Nanoparticle Preparation

Chitosan nanoparticles were prepared according to Calvo et al. [19]. Briefly, water-soluble chitosan was dissolved in aqueous solution. Nanoparticles were formed spontaneously upon addition of 2 mL of the sodium tripolyphosphate (TPP in sodium citrate, 238503, Sigma-Aldrich) aqueous solution (10%) to 100 mL of the chitosan acidic solution (448869, Sigma-Aldrich, low molecular weight, 75–85% deacetylation, 1 mg/mL, w/v) under magnetic stirring at room temperature. The pH of chitosan solution varied between 3.0 and 5.0. PEG (10 mg/mL, 1 mL) was dissolved in the chitosan solution after the addition of the TPP solution. Nanoparticles were isolated by ultracentrifugation (25,000 g, 15 min) and then resuspended in water by manual shaking.

### 2.2. Characterization of Chitosan Nanoparticles

The morphological examination of the chitosan nanoparticles was performed by transmission electron microscopy at an accelerating voltage of 100 kV (TEM, JEM 1200CX II, JEOL). The sample was stained with 2% (w/v) phosphotungstic acid and placed on copper grids for viewing by TEM.

The particle size and size distributions of the chitosan nanoparticles were performed by particle size analyzer (90 plus particle sizer, Brookhaven Instruments Corp., USA). For the particle size analysis, each sample was diluted to the appropriate concentrating with filtered distilled water. Each analysis lasted 2 min and was performed at 25°C with an angle detection of 90°.

Measurement of the zeta potential of nanoparticles was performed by Zeta plus 90 particle sizer (Brookhaven Instruments Corp., USA) with a 5 mW He-Ne laser (*λ* = 663 nm). The zeta potential values were calculated from the mean electrophoretic mobility values using Smoluchowski's equation.

### 2.3. Cell Culture

HepG2 (liver tumor cell) and CCL-13 (liver normal cell) cells were maintained at 37°C and 5% CO_2_ in RPMI 1640 medium (Gibco, USA) supplemented with 10% fetal bovine serum (FBS, Hyclone Laboratories, Logan, UT), 1% penicillin/streptomycin (Gibco, Grand Island, NY, USA), and 44 mM NaHCO_3_ (Sigma, USA). After three days, the cells were washed with serum-free RPMI 1640 medium and incubated with the serum-free medium containing chitosan nanoparticles at concentrations of 1 to 5 *μ*g/mL for 12 h.

### 2.4. BrdU and LDH Assay

CCL-13 and HepG2 cells were seeded in a sterile 96-well tissue culture plate at 2 × 10^5^ cells/mL in 100 *μ*L/well of appropriate cell culture media with chitosan nanoparticles. The cell proliferation was determined by bromodeoxyuridine assay (BrdU Cell Proliferation Assay, Millipore, USA). The cytotoxicity of chitosan nanoparticles was evaluated* in vitro* using the lactate dehydrogenase assay (LDH Cytotoxicity Assay, ScienCell Research Laboratories, USA). These assays were performed according to the manufacturers' instructions. The absorbance values were measured by an ELISA reader (Multiskan EX, Thermo scientific, Vantaa, Finland, reference wavelength: 450 nm).

### 2.5. Cell Morphology

For cell morphologies of HepG2 and CCL-13 before and after incubation with chitosan nanoparticles, the cell live images were observed with a microscope equipped with fluorescence light source (FLoid cell fluorescence imaging Station, Invitrogen), and the cell micrographs were taken with a CCD camera.

### 2.6. Protein Sample Preparation

After incubation with chitosan nanoparticles, the HepG2 and CCL-13 cells were lysed by cell lysis buffer (3500-1, Epitomics, Inc, USA), and cell lysates were centrifuged at 1500 ×g for 10 min at 4°C. The supernatants were flitted by 0.8 *μ*m filter and the protein concentrations were adjusted to 1 mg/mL by 25 mM ammonium bicarbonate.

Cell lysates samples (100 *μ*L) were transferred into the 1.5 mL Eppendorf tubes and incubated at 37°C for 3 h after mixing with 25 *μ*L of 1 M dithiothreitol (DTT, USB Corporation, 15397). Then cell lysates samples were reduced and alkylated in the dark at room temperature for 30 min after addition of 25 *μ*L of 1 M iodoacetamide (IAA, Amersham Biosciences, RPN6302V) in 25 mM ammonium bicarbonate. Approximately 10 *μ*L of 0.1 *μ*g/*μ*L modified trypsin digestion buffer (Trypsin Gold, Mass Spectrometry Grade, V5280, Promega, WI, USA) in 25 mM ammonium bicarbonate was added to the cell lysates samples, and the cell lysates samples were incubated at 37°C for at least 12 h in a water bath. Two microliter of formic acid was added to each sample before mass spectrometric analysis for protein identification.

### 2.7. Proteomic Analysis

The complex peptide mixtures were separated by RP-nano-UPLC-ESI-MS/MS. The protein tryptic digests were fractionated using a flow rate of 400 nL/min with a nano-UPLC system (nanoACQUITY UPLC, Waters, Milford, MA) coupled to an ion trap mass spectrometer (LTQ Orbitrap Discovery Hybrid FTMS, Thermo, San Jose, CA) equipped with an electrospray ionization source. For RP-nano-UPLC-ESI-MS/MS, a sample (2 *μ*L) of the desired peptide digest was loaded into the reverse phase column (Symmetry C18, 5 *μ*m, 180 *μ*m × 20 mm) by an autosampler. The RP separation was performed using a linear acetonitrile gradient from 99% buffer A (100% D.I. water/0.1% formic acid) to 85% buffer B (100% acetonitrile/0.1% formic acid) in 100 min using the micropump. The separation is performed on a C18 microcapillary column (BEH C18, 1.7 *μ*m, 75 *μ*m × 100 mm) using the nanoseparation system. As peptides eluted from the microcapillary column, they were electrosprayed into the ESI MS/MS with the application of a distal 2.1 kV spraying voltage with heated capillary temperature of 200°C. Each cycle of one full scan mass spectrum (m/z 400–2000) was followed by four data dependent tandem mass spectra with the collision energy set at 35%.

### 2.8. Database Search

For protein identification, Mascot software (Version 2.2.1, Matrix Science, London, UK) was used to search the Swiss-Prot human protein sequence database. Positive protein identifications were defined when Mowse scores greater than 100 were considered significant (*P* < 0.05). Proteins were annotated by similar searches using UniProtKB/Swiss-Prot databases. The protein-protein interaction pathways were performed by String 9.1 Web software.

### 2.9. Statistical Analysis

All calculations used the SigmaStat statistical software (Jandel Science Corp., San Rafael, CA). All statistical significances were evaluated at 95% of confidence level or better. Data are presented as mean ± standard error.

## 3. Results and Discussions

### 3.1. Size, Zeta Potential, and Morphology of Chitosan Nanoparticles

**Figure 1:**
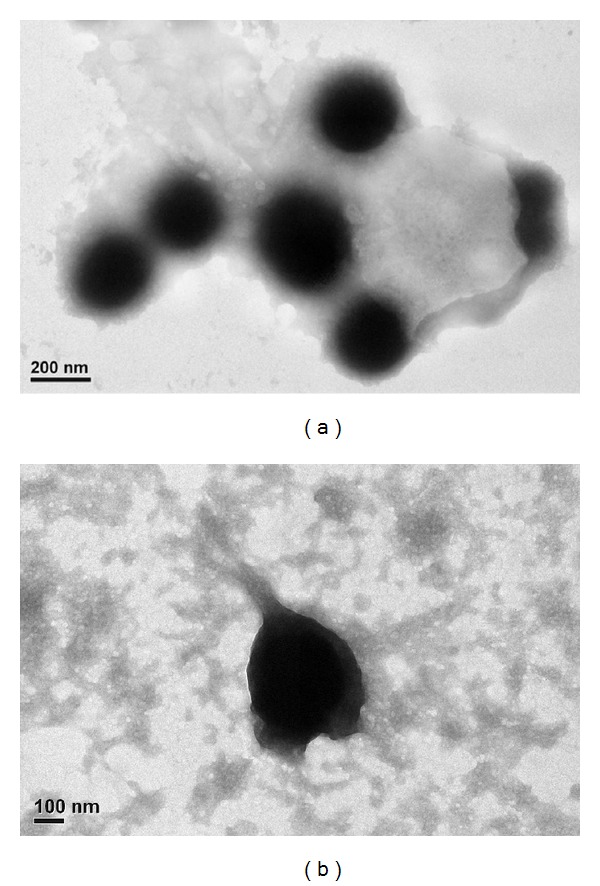
The morphological examination of the chitosan nanoparticles was performed by transmission electron microscopy at an accelerating voltage of 100 kV. The mean of diameter is around 236.3 nm.

As determined by particle size and zeta potential analyzers, the average size of the chitosan nanoparticle was 224.6 ± 11.2 nm, and the average zeta potential was +14.08 ± 0.7 mV in phosphate-buffered saline. The size and surface morphology of chitosan nanoparticles was shown in Figure 1. The TEM image displays the clear spherical morphology of the chitosan nanoparticles having a mean diameter of chitosan nanoparticles about 236.3 nm as shown. Zeta potential is the surface charge of nanoparticles and can influence the nanoparticle stability in suspension through the electrostatic repulsion between nanoparticles. In this study, the surface charge of chitosan nanoparticles was positive, according to the protonation of NH_2_ functional groups of glucosamine units to NH_3_^+^ ion.

### 3.2. In Vivo Chitosan Nanoparticle Uptake

**Figure 2:**
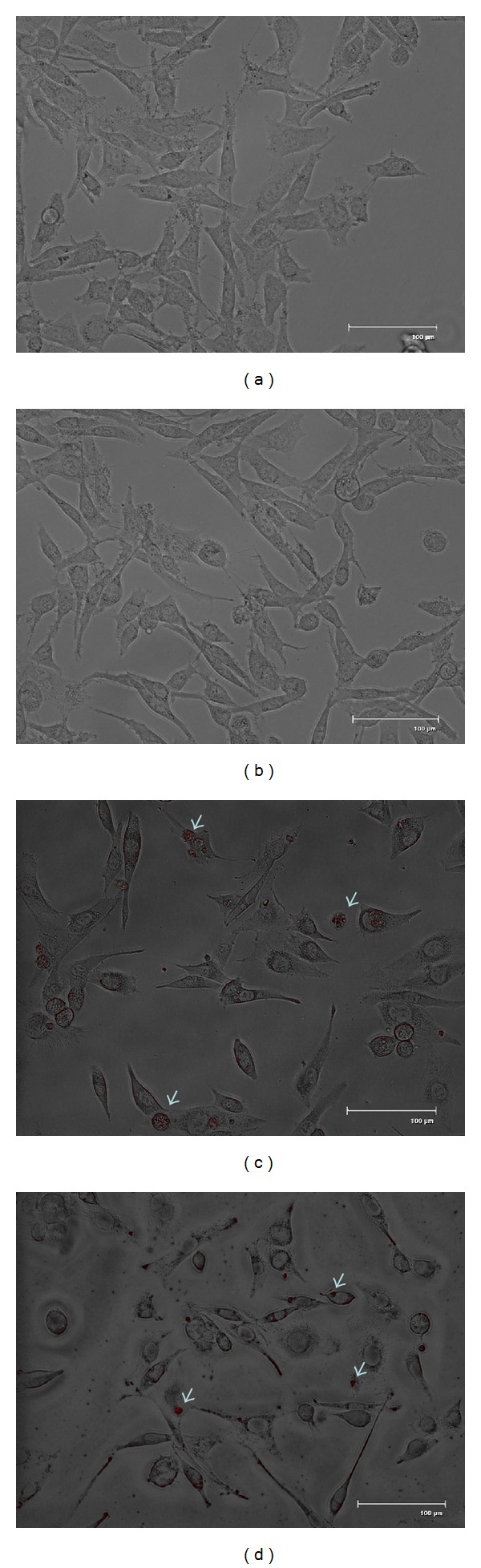
The live fluorescent images of chitosan nanoparticles (red) taken by CCL-13 cells (a, c) and HepG2 cells (b, d). (a) and (b): cells without chitosan nanoparticles; (c) and (d): cells with chitosan nanoparticles at a concentration of 5 *μ*g/mL for 12 h at 37°C; the red fluorescence was localized near by the cell nuclei. Those images represented merged images of DIC and red fluorescence. (600x, scale bar: 100 *μ*m).

*In vitro* uptake of chitosan nanoparticles was evaluated by fluorescence microscopy. CCL-13 and HepG2 cells were incubated with the growth medium containing chitosan nanoparticles at a concentration of 5 *μ*g/mL for 12 h at 37°C, respectively. As expected, no red fluorescence signals were detected in sections of the cells without chitosan nanoparticles (Figure 2(a): CCL-13 and Figure 2(b): HepG2, 600x, scale bar: 100 *μ*m). Cell micrographs from chitosan nanoparticles treated CCL-13 and HepG2 cells reveled red fluorescence localized near by the cell nuclei (Figure 2(c): CCL-13 and Figure 2(d): HepG2, 600x, scale bar: 100 *μ*m). In some HepG2 cells, the fluorescence was also localized in the cytoplasm.

### 3.3. Cytotoxicity of Chitosan Nanoparticle

To examine the cytotoxicity, HepG2 and CCL-13 cells were incubated with chitosan nanoparticles for 12 h. LDH and BrdU assays are quantitative colorimetric assays for mammalian cell survival and cell proliferation.

**Figure 3:**
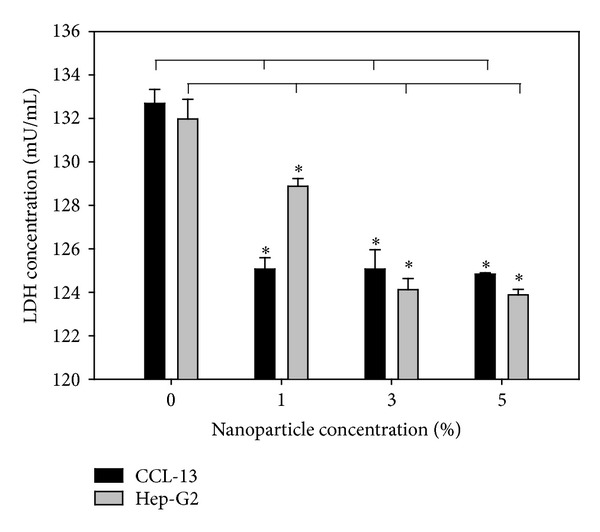
LDH test of chitosan nanoparticle (CSNP) effects on Chang and HepG2 cells (*N* = 6, mean ± standard error, *t*-test, *P* < 0.05).

**Figure 4:**
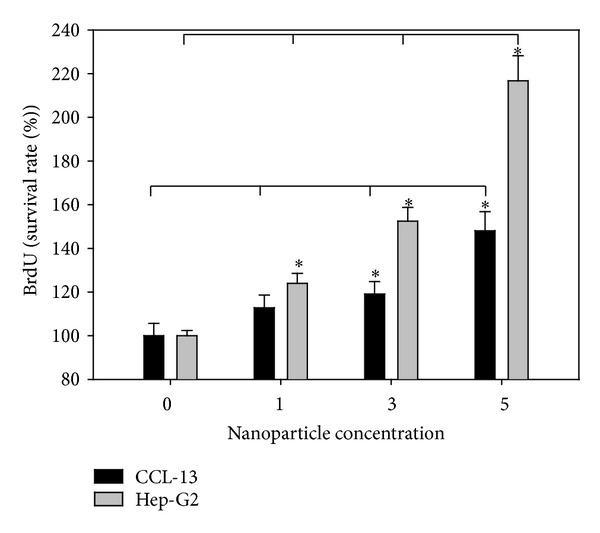
Proliferation (BrdU) test of chitosan nanoparticle (CSNP) effects on Chang and HepG2 cells (*N* = 6, mean ± standard error, *t*-test, *P* < 0.05).

The cell death is assayed by the quantification of a stable cytoplasmic enzyme activity, LDH, which was released into the cell culture supernatant upon cell death and damage of the cytoplasmic membrane. BrdU is integrated into newly synthesized DNA strands of actively proliferating cells. Following partial denaturation of double stranded DNA, BrdU is detected immunochemically allowing the assessment of the number of cells which are synthesizing DNA. As shown in Figure 3, the LDH concentrations were decreased and observed between the groups treated with different concentration of chitosan nanoparticle and control (*P* < 0.05, *N* = 6). Compared with the control, the BrdU was upregulated in the groups treated with chitosan nanoparticle with significantly increase (Figure 4, *P* < 0.05, *N* = 6). Those results indicate that the chitosan nanoparticle was no significant cytotoxicity observed; in addition, the chitosan nanoparticle improved the cell growth and proliferation. In previous studies, it was indicated that the metal or metal oxide nanoparticles were with high cell toxicity [22–24]. Unlike metal or metal oxide nanoparticles, the chitosan nanoparticle was nontoxic. The cell growth and survival rate were increased with the higher concentration of chitosan nanoparticles. As shown in Figures 3 and 4, the chitosan nanoparticle was enhanced cell growth in a dose dependent manner.

### 3.4. Proteomic Analysis of Cell Response to Chitosan Nanoparticle

To investigate the effect of chitosan nanoparticle on liver normal and tumor cells, a proteomic approach, such as RP-nano-UPLC-ESI-MS/MS analysis, was utilized to analyze cell lysates. The traditional methods use individual antibodies to evaluate the cell response to nanoparticles, but the proteomic approach can be used to analyze an enormous number of proteins simultaneously. In this study, HepG2 and CCL-13 cells were incubated with chitosan nanoparticles. After 12 h, the HepG2 and CCL-13 cells were lysed, and the cell lysates were digested by trypsin; generating tryptic peptides was subsequently analyzed by RP-nano-HPLC-ESI-MS/MS, respectively. The RP-nano-HPLC-ESI-MS/MS approach is perhaps the most representative method in proteome researches. The fragmentation spectra obtained by the RP-nano-HPLC-ESI-MS/MS analysis in gradient detection mode were compared with a nonredundant protein database using Mascot software. All Mascot results were visually confirmed. In addition, the criterion requires a readily observable series of at least four y ions for an identified peptide [25]. When a protein was identified by three or more unique peptides, no visual assessment of spectra was conducted and the protein was considered to be present in the sample.

In this study, more than one hundred proteins were identified and most of these were identified at the minimal confidence level, which was only one unique peptide sequence matched. Experimental results reported a total of eight protein identifications with higher confidence levels (at least three unique peptide sequences matched) and exhibited significant differences between the chitosan nanoparticle treated and nontreated cells. Those cell lysate proteins were involved in cell growth, differentiation, division, cycle regulation (6 in HepG2 cells and 2 in CCL-13 cells).

**Figure 5:**
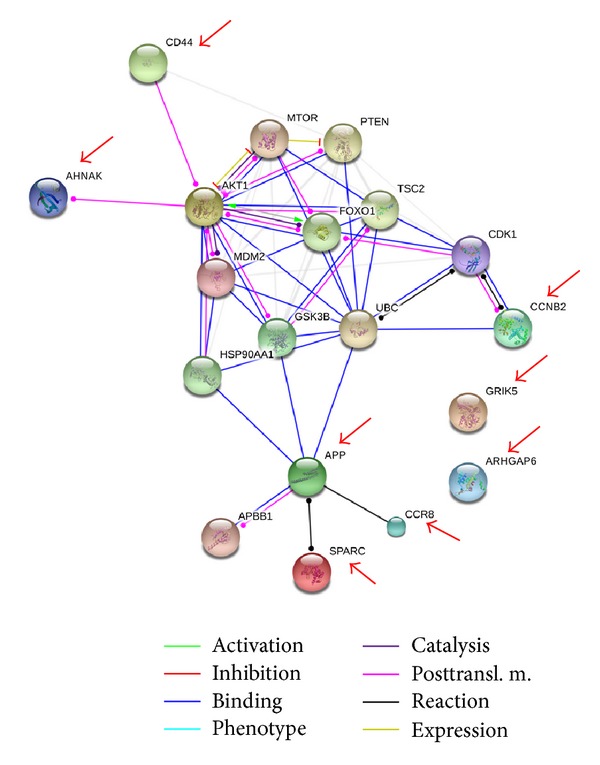
The protein-protein interaction pathways were performed. Proteins identified in this study were marked by arrows. The CD44 and APP can turn on the PI3K/AKT/mTOR pathway, which is responsible for the proliferation and is required for survival of the majority of cells.

A summary of the protein identifications achieved is shown in Table 1. For the eight protein identifications, six proteins were positively identified as Amyloid beta A4 protein (APP, P05067), C-C chemokine receptor type 8 (CCR8, P51685), CD44 antigen (CD44, P16070), G2/mitotic-specific cyclin-B2 (CCNB2, O95067), Neuroblast differentiation-associated protein (AHNAK, Q09666), and Osteonectin/SPARC protein (SPARC, P09486) in HepG2 cell lysate. Two proteins, Glutamate receptor ionotropic, kainate 5 (GRIK5, Q16478), and Rho GTPase-activating protein 6 (ARHGAP6, O43182) were found in the CCL-13 cell lysate. The protein-protein interaction pathways were performed by String 9.1 Web software. Eight proteins identified in this study were marked by red arrows (Figure 5).

GRIK5 and ARHGAP6 were not detected in the HepG-2 cells but in CCL-13 cells. GRIK5 gene belongs to the kainate family of glutamate receptor in the cerebellum and the suprachiasmatic nuclei (SCN) of the hypothalamus, which is composed of four subunits and function as ligand-activated ion channels [26]. It is the predominant excitatory neurotransmitter receptor in the brain of mammalian and is involved in the neurophysiologic processes, such as the transmission of light information from the retina to the hypothalamus.

ARHGAP6 is a regulatory protein which can bind to activated G proteins and stimulate their GTPase activity. Regulation of G proteins is important because these proteins are involved in a variety of important cellular processes. It may result in transduction of signaling from the G protein-coupled receptor for a variety of signaling processes like hormonal signaling and involve in processes like cellular trafficking and cell cycling [27]. In this study, GRIK5 and ARHGAP6 were identified in the CCL-13 cells after chitosan nanoparticle treatment. However, the analyses of protein functions and protein-protein interaction pathways did not show the serious effect in CCL-13 cells.

In this study, the main finding of chitosan nanoparticle treated cells is the chitosan nanoparticles enhancing the PI3K/AKT1/mTOR pathway in Hep-G2 cells which may result in tumor metastasis. There were six proteins identified in HepG-2 cell lysate samples, which may relate to metastasis and cell proliferation. Using the protein-protein interaction pathway analysis, those six proteins were related to the PI3K/AKT1/mTOR pathway (Figure 5). The CD44 can turn on the PI3K/AKT1/mTOR pathway, which is responsible for the proliferation and is required for survival of the majority of cells. The PI3K/AKT1/mTOR pathway can also be turned on by GSK3B when GSK3B is bound with APP and SPARC.

The mammalian target of rapamycin pathway (mTOR pathway, also known as FRAP, RAFT1, and RAP1 pathway) has been identified as a key kinase acting downstream of the activation of phosphoinositide-3-kinase (PI3K) [28]. The PI3K/AKT1/mTOR pathway is responsible for the proliferation and is required for survival of the majority of cancer cells. The hypothesis of the mTOR pathway is acting as a master switch of cellular catabolism and anabolism, thereby determining whether cells grow and proliferate of tumor cells [29, 30]. Activation of PI3K/AKT1/mTOR signaling through mutation of pathway components as well as through activation of upstream signaling molecules occurs in a majority of cancer cells contributing to deregulation of proliferation, resistance to apoptosis, and changes in metabolism characteristic of transforming cells [31].

## 4. Conclusion 

As previous reports have indicated that the chitosan nanoparticle was nontoxic for cell lines and appropriate as a drug carrier in micro capsule. In this study, the experimental results showed that it is a dose dependent manner to enhance cell growth. However, according to the proteomics analysis, chitosan nanoparticle induced the liver cancer cell to secret several proteins which may active or enhance PI3K/AKT1/mTOR pathway. The PI3K/AKT1/mTOR pathway was related to the cancer call metastasis and proliferation. Although there are no direct evidences to prove the relevance between chitosan nanoparticle and PI3K/AKT1/mTOR pathway in our study, it is still worth to be considered, especially as an anticancer drug delivery system.

## Figures and Tables

**Table 1:** The eight unique proteins identified by the higher confidence level (at least three unique peptide sequences matched) with significant difference between CCL-13 and HepG2 cells incubation with chitosan nanoparticles in this study.

Cell	Swiss-Prot Number	Protein Name	Subcellular location	MW (Da)	Score	Queries	pI	Molecular function	Sequence coverage	Peptide
CCL-13	Q16478	Glutamate receptor, ionotropic kainate 5 (GRIK5)	Cell membrane	109195	82	9	8.54	extracellular-glutamate-gated ion channel activity kainate selective glutamate receptor activity	28%	K.VSTIIIDANASISHLILRK.A + Deamidated (NQ); 2 Phospho (ST) R.LQYLRFASVSLYPSNEDVSLAVSRILK.S + 2 Deamidated (NQ) K.VGPEETPRLQYLR.F R.LNCNLTQIGGLLDTK.G + 2 Deamidated (NQ); Phospho (ST) R.LNCNLTQIGGLLDTK.G + 3 Deamidated (NQ); Phospho (ST) R.RLNCNLTQIGGLLDTK.G + 2 Deamidated (NQ); Phospho (ST) K.VSTIIIDANASISHLILRK.A + Deamidated (NQ); 2 Phospho (ST) M.PAELLLLLIVAFASPSCQVLSSLR.M + Deamidated (NQ) -.MPAELLLLLIVAFASPSCQVLSSLRMAAILDDQTVCGR.G + Carbamidomethyl (C); Deamidated (NQ); Oxidation (M); 2 Phospho (ST)
	O43182	Rho GTPase-activating protein 6 (ARHGAP6)	Cytoplasm	105882	72	13	7.11	Rho GTPase activator activity SH3/SH2 adaptor activity phospholipase activator activity	29%	R.EQQVTQK.K K.DPGMTGSSGDIFESSSLR.A + 4 Phospho (ST) -.MSAQSLLHSVFSCSSPASSSAASAK.G + Deamidated (NQ); Oxidation (M); 3 Phospho (ST) R.EQQVTQKKLSSANSLPAGEQDSPR.L + 2 Phospho (ST) K.DASDFVASLLPFGNK.R + Deamidated (NQ); Phospho (ST) R.LRSVPIQSLSELERAR.L + Deamidated (NQ) R.LRSVPIQSLSELERAR.L + Phospho (ST) K.MTSLNLATIFGPNLLHKQK.S + Deamidated (NQ); Oxidation (M) K.DPGMTGSSGDIFESSSLR.A + 4 Phospho (ST) R.TQAAAPATEGR.A + Deamidated (NQ); Phospho (ST) R.TQAAAPATEGR.A + Deamidated (NQ); Phospho (ST) K.DPGMTGSSGDIFESSSLR.A + 4 Phospho (ST) R.HSSTDSNKASSGDISPYDNNSPVLSER.S + Deamidated (NQ); 6 Phospho (ST)

HepG2	P05067	Amyloid beta A4 protein (APP)	Membrane	86888	49	4	4.73	DNA binding heparin binding peptidase activator activity serine-type endopeptidase inhibitor activity transition metal ion binding	19%	R.LNMHMNVQNGK.W + Deamidated (NQ); 2 Oxidation (M) K.WDSDPSGTKTCIDTK.E + 5 Phospho (ST) K.GAIIGLMVGGVVIATVIVITLVMLKK.K + Oxidation (M); Phospho (ST) R.ALEVPTDGNAGLLAEPQIAMFCGR.L + 2 Deamidated (NQ); Oxidation (M); Phospho (ST)
	P51685	C-C chemokine receptor type 8 (CCR8)	Cell membrane	40817	47	4	8.66	C-C chemokine receptor activity chemokine receptor activity coreceptor activity	12%	R.ESCEKSSSCQQHSSR.S + Carbamidomethyl (C); Deamidated (NQ); 3 Phospho (ST) R.ESCEKSSSCQQHSSR.S + Carbamidomethyl (C); 2 Deamidated (NQ); 5 Phospho (ST) R.ESCEKSSSCQQHSSR.S + Carbamidomethyl (C); 2 Deamidated (NQ); 5 Phospho (ST) R.ESCEKSSSCQQHSSR.S + Carbamidomethyl (C); 2 Deamidated (NQ); 5 Phospho (ST)
	P16070	CD44 antigen (CD44)	Membrane	81487	60	3	5.13	collagen binding hyaluronic acid binding hyalurononglucosaminidase activity	4%	R.YGFIEGHVVIPR.I K.ALSIGFETCRYG FIEGHVVIPR.I K.EQWFGNRWHEGYR.Q
	O95067	G2/mitotic-specific cyclin-B2 (CCNB2)		45253	58	3	9.21		16%	R.KKLQLVGITALLLASK.Y K.VPVQPTKTTNVNKQLKPTASVKPVQMEK.L + Deamidated (NQ); Oxidation (M); Phospho (ST) K.AQNTKVPVQPTKTTNVNK.Q + 3 Deamidated (NQ); 2 Phospho (ST)
	Q09666	Neuroblast differentiation-associated protein (AHNAK)	Nucleus	628699	39	7	5.8		12%	K.GPKFKIPEMHLK.A K.FKMPSMNIQTHK.I + Deamidated (NQ); Oxidation (M); Phospho (ST) K.MDAEVPDVNIEGPDAKLK.G + Deamidated (NQ) K.KSRLSSSSSNDSGNK.V + 2 Deamidated (NQ); 6 Phospho (ST) K.MKLPQFGISTPGSDLHVNAK.G K.VHAPGLNLSGVGGKMQVGGDGVK.V + Deamidated (NQ); Oxidation (M); Phospho (ST) R.AGAISASGPELQGAGHSKLQVTMPGIKVGGSGVNVNAK.G + 2 Deamidated (NQ); Oxidation (M); 4 Phospho (ST)
	P09486	Osteonectin/ SPARC protein (SPARC)	Secreted	34610	89	3	4.73	calcium ion binding collagen binding extracellular matrix binding	4%	R.LEAGDHPVELLAR.D K.HGKVCELDENNTPMCVCQDPTSCPAPIGEFEK.V + 3 Carbamidomethyl (C) R.APLIPMEHCTTR.F
